# A Case Report of Posterior Communicating Artery Aneurysm Presenting as Cranial Nerve 3 Palsy in a Young Female Patient with Migraines

**DOI:** 10.21980/J8QW83

**Published:** 2021-01-15

**Authors:** Colin Danko, Dustin Williams

**Affiliations:** * University of Texas Southwestern Medical Center, Department of Emergency Medicine, Dallas, TX

## Abstract

This is a case report of a 33-year-old female with a history of migraines who presented with her typical migraine symptoms for three days. However, she also began to notice diplopia and drooping of her right eyelid, which were symptoms she had never had in the past with migraines. MR angiography showed a 4x3x5 mm posterior communicating artery aneurysm that was likely compressing the right third cranial nerve. She was taken to the operating room by neurosurgery and underwent clip ligation of the aneurysm with improvement in symptoms. It is important to always assess for new signs and symptoms in patients presenting to the Emergency Department who are complaining of their “typical” migraine symptoms because this may indicate previously undiagnosed pathology that will require intervention to prevent negative outcomes.

**Topics:**

Cerebral aneurysm, cranial nerve palsy, ptosis.

**Figure:**
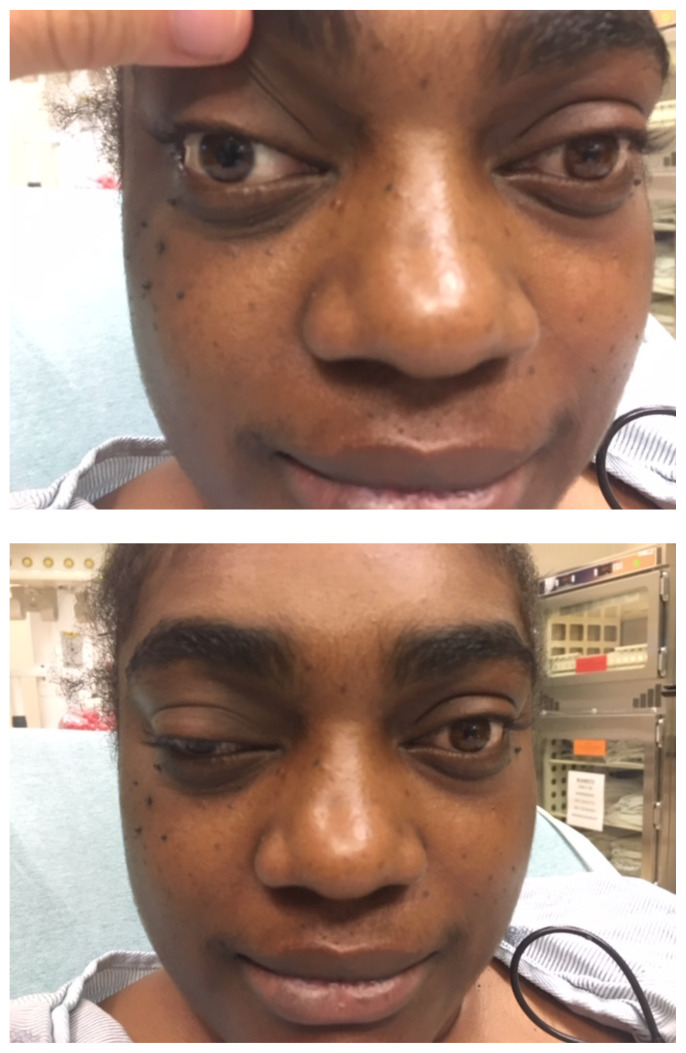


## Introduction

Headaches are one of the most common chief complaints encountered in the emergency department (ED), making up approximately 3–4% of all ED visits.^1^,^2^ Headaches are often related to primary headache syndromes such as tension headaches, migraines, cluster headache, and others.^1^ Patients with these types of headaches usually require only symptomatic treatment and are discharged. However, there are several underlying pathophysiological processes that can also cause headaches and are more dangerous.^1^,^2^ It is the job of the emergency medicine provider to not only provide effective symptomatic treatment, but to ensure there is no underlying, life-threatening cause.^1^

This case involves a patient who presented with a complaint of her “typical migraine.” However, she also noted new-onset diplopia and right sided ptosis. A patient with a headache who has additional complaints, such as a new neurological deficit, should be a red flag that a secondary cause may be producing the headache.

## Presenting concerns and clinical findings

A 33-year-old female with past medical history of migraines, hypertension, and a previously diagnosed pericallosal lipoma presented to the emergency department with right-sided, throbbing headache for the last three days. This was associated with photophobia, phonophobia and nausea with no emesis. Initially, this presentation was similar to migraines she had in the past. However, she began to notice that her right eyelid was drooping and she complained of vertical and horizontal diplopia that corrected with rightward gaze. She denied any recent head trauma, focal weakness, numbness, slurred speech, or other neurological deficits.

## Significant findings

Physical exam revealed a right pupil that was dilated compared to the left pupil, though both pupils were reactive. The patient also had impaired medial gaze on the right and ptosis of the right eyelid. Exam was otherwise unremarkable.

## Patient course

Initial computed tomography (CT) of the head was unremarkable, aside from remonstration of previously seen pericallosal lipoma. Magnetic resonance imaging (MRI) and magnetic resonance angiography (MRA) of the brain showed a right posterior communicating artery aneurysm measuring 4x3x5mm projecting inferiorly into the right cavernous sinus with probable mass effect on the right third cranial nerve. The patient was admitted to the neurology intensive care unit for monitoring and blood pressure control overnight. She was taken to the operating room by neurosurgery the following morning for craniotomy and clip ligation of the aneurysm, with subsequent improvement in her 3rd cranial nerve palsy at clinic follow up.

## Discussion

Headaches are one of the most common presenting complaints encountered in the Emergency Department, and while they are often caused by a benign, primary headache syndrome such as migraine, tensions headaches, and others, it is vital that the emergency physician closely evaluate for subtle signs and symptoms that suggest a dangerous underlying cause.^2^ Taking a thorough patient history and conducting a comprehensive physical exam, with special attention to the neurologic exam, will allow the physician to detect subtle red flags that necessitate further workup, including advanced imaging. Such red flags include acute onset of a severe headache, age greater than 50, pregnancy, HIV or other immunocompromised state, fever, known malignancy, anticoagulant use, and new neurologic deficits, such as the third cranial nerve palsy seen in this case^2^.

The third cranial nerve provides motor innervation to several muscles of the eye including the superior, inferior, and medial rectus muscles, the superior oblique muscle, and the levator complex.^3^ It additionally supplies parasympathetic innervation to the iris and ciliary body^3^. Because of this, physical exam in patients with third cranial nerve palsy classically shows a pupil that is “down and out” with ptosis on the affected side.^4^ In cases of external compression of the nerve, the pupil will be fixed and dilated, given that the parasympathetic fibers that control pupillary dilation are peripherally located. In cases of palsy caused by ischemia, the pupil will not be dilated because the vasculature is centrally located, sparing the parasympathetic fibers.^4^ Patients with third cranial nerve palsy will often present with complaints of binocular diplopia that corrects with lateral gaze and may notice a drooping eyelid on the affected side.^5^ There are many causes of third cranial nerve palsy, including ischemia (often associated with diabetes and hypertension), intracranial trauma leading to uncal herniation, idiopathic, and compression due to intracranial aneurysms.^4^,^5^,^6^ Aneurysms causing compression of the third cranial nerve most commonly arise from the junction of the posterior communicating artery and the internal carotid artery, but may also occur along the basilar artery.^7^ The previous gold standard method of detecting such aneurysms was cerebral catheter angiography, though the effectiveness of MRA and CT angiography have significantly improved, with respective sensitivities of 97% and 95% for detecting aneurysms >5 mm in size.^7^,^8^ Intervention is recommended for asymptomatic aneurysms greater than 7 mm in size because the risk of rupture has been found to be greater than 2% and increases with aneurysm size.^8^ However, all symptomatic intradural aneurysms should be considered for urgent intervention, regardless of size.^8^ Studies have found that intravascular interventions, such as endovascular coiling, are associated with lower morbidity and mortality when compared with surgical clipping.^9^

## Supplementary Information




