# Supplementary figures and images for: A Case Report of Posterior Communicating Artery Aneurysm Presenting as Cranial Nerve 3 Palsy in a Young Female Patient with Migraines
Source: J Educ Teach Emerg Med. 2021 Jan 15;6(1):V5–8. doi: 10.21980/J8QW83 (PMC10332754; doi:10.21980/J8QW83)

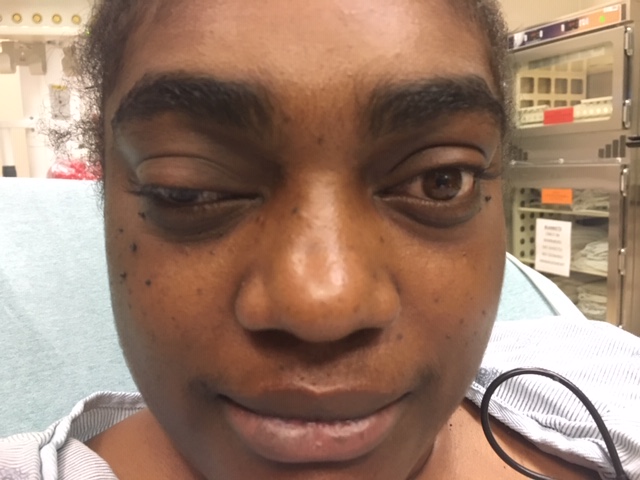

Supplement: Supplementary file 1 [file jetem-6-1-v5-supp1.jpg]

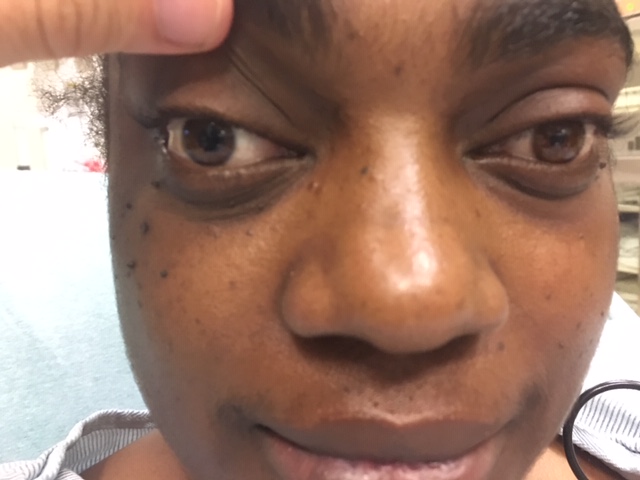

Supplement: Supplementary file 2 [file jetem-6-1-v5-supp2.jpg]
